# The Microscope

**Published:** 1869-01

**Authors:** R. C. Kendall


THE MICROSCOPE: *Its Use in Entomology—in the First American Discovery of the "Trichina Spiralis.*

"MYSTERIES OF THE MICROSCOPE.—Not that there is any especial hidden mystery in the innocent-looking, modest little instrument that presents objects to us as they really are, making huge monsters out of mere mites, and as often presenting most magnificent animals in what, to the unaided eye appears an uncouth atom. The mystery is of the microscope. Its power, to our intelligence, as at present educated, is unintelligible, and would be magical, but that we know the microscope to be innocent of the black art, and the maker only a man like ourselves—a trifle more clever, perhaps, but not a mite of a magician. So much of thought is involved by the advent of a red mite upon the edge of the white sheet now under the point of my pen, and the ruby dot—a mere point to the naked eye—hurrying over the white field, a perfect crimson streak. If a man were to run at that rate, according to bulk, he would get over the ground about a thousand miles an hour. and race entirely round the world in a day and night, with three hours left for rest and refreshment.

"Arresting the atomic red runaway, and clapping him under my SEMPER PARATUS Craig Microscope, in an instant I had under my eye a wonder, a bright crimson bird, wingless like the penguin, but perfect in proportions, and of exquisite beauty; its downy plumage brilliantly bright; its six perfect bird legs, three set on either side. I saw there the secret of the rapid race. Fancy a turkey gobbler with six legs, each one putting in its quota of speed! Wouldn't the old fellow outrun a hurricane? Then there are the five white delicate toes, more like a fair lady's fingers, to each foot; black, lustrous eyes; and beak like that of the great 'war eagle'—all harmonious; but strange—very wonderful—mysterious—the manner in which that single bit of clear glass metamorphoses the tiny red mite into a great magnificent bird! There, go out with you, and go your way, diminish to a red atom, almost infinitessimal again! Scud—scatter, crimson speck, and leave me to my say of my magnifying miracle.

"Before I was the proprietor of this Craig glass, for which I paid $2.50, I had for ten years used a French instrument, which cost me, I think, $55.00, of feebler power, and less reliable. With the French 'Cressaix,' I searched long and fruitlessly for the 'trichina spiralis,' that savants guessed was in our American pork. With the $2.50 Craig I laid hold of it plainly and positively at the second trial. That was two years ago this month. Is it recorded that any one had discovered the pork pests earlier than that date? If not, then they were first found under an American microscope; and so much for the skill and ingenuity of American mechanism.

"For the farmer and fruit-grower, especially, these simple, practical instruments are invaluable; and, to their children, a source of education, amusement, and real instructive pleasure, of which they will never grow weary. A bright little girl of ten years, daughter of a farmer friend, to whom I loaned mine, actually acquired a fuller and more correct knowledge of half a hundred insect inhabitants of her neighborhood, in six weeks' practice with the microscope, than a professed entomologist, principal of a neighboring seminary, had acquired in thirty years of study."—*From the American Farmer. Dr. R. C. Kendall.*

